# Bored with boredom? Trait boredom predicts internet addiction through the mediating role of attentional bias toward social networks

**DOI:** 10.3389/fnhum.2023.1179142

**Published:** 2023-09-01

**Authors:** Loreta Cannito, Irene Ceccato, Eugenia Annunzi, Alessandro Bortolotti, Eleonora D’Intino, Rocco Palumbo, Claudio D’Addario, Alberto Di Domenico, Riccardo Palumbo

**Affiliations:** ^1^Department of Humanities, University of Foggia, Foggia, Italy; ^2^Center for Advanced Studies and Technology (CAST), Chieti, Italy; ^3^Department of Psychological, Health and Territorial Sciences, “G. d’Annunzio” University of Chieti-Pescara, Chieti, Italy; ^4^Department of Neuroscience, Imaging and Clinical Sciences, “G. d’Annunzio” University of Chieti-Pescara, Chieti, Italy; ^5^Faculty of Bioscience and Technology for Food, Agriculture and Environment, University of Teramo, Teramo, Italy; ^6^Department of Clinical Neuroscience, Karolinska Institutet, Stockholm, Sweden

**Keywords:** information processing, attention paradigms, social networks, problematic internet use, internet addiction, dot-probe task

## Abstract

Internet addiction is an emerging issue, impacting people’s psychosocial functioning and well-being. However, the prevalence and the mechanisms underlying internet misuse are largely unknown. As with other behavioral addiction disorders, the increase and persistence of internet addiction may be favored by negative affect such as boredom. In this study, we examined the role of boredom susceptibility, as a personality trait, in predicting the risk of internet addiction. Furthermore, we analyzed the attentional mechanisms that may exacerbate dysfunctional internet behaviors. Specifically, we assessed the mediating role of attentional bias toward social media cues on the relation between boredom susceptibility and internet addiction. Sixty-nine young adults were administered a dot-probe task assessing internet-related attentional bias (AB) and questionnaires measuring internet addiction (IAT) and boredom susceptibility (BS-BSSS). Correlation and *t*-test analyses confirmed that the tendency to experience boredom and selective attention toward social network information was related to internet addiction. Furthermore, the mediation model indicated that AB fully explains the link between BS-BSSS and IAT. The study highlighted the crucial role of selective attentional processing behind internet addiction. The current results are useful for both researchers and clinicians as they suggest that intervention programs for internet addiction should include strategies to cope with dysfunctional cognitive processes.

## Introduction

Along with the beneficial improvements that the internet has brought about in society, several issues related to problematic internet usage and addiction have also emerged. According to a large body of research, this dysfunctional condition can have a significant impact on the quality of “real” life by negatively affecting time spent in social interactions (Enez Darcin et al., 2016), restricting one’s capacity to fulfill commitments at the professional and academic levels (Young, 1996; Annunzi et al., 2022), or even interfering with time spent engaging in personal interests (Hellström et al., 2012; Rehbein and Baier, 2013). Within the broader category of internet addiction, the most heavily studied phenomena are certainly betting, gaming, and social network addictions (Petry, 2015; Calluso et al., 2020; Cannito et al., 2022a). However, partly due to the more recent spread of social networks, much more evidence is available on gaming addiction although it has recently been reported that social network addiction occurs more frequently in the population and it is equally, if not more, associated with psychosocial difficulties (Burén et al., 2021). A massive increase in social network addiction was reported alongside the spread of mobile hardware (smartphones and tablets) as it made it possible to be always connected (Schou Andreassen and Pallesen, 2014). From the emergence of this phenomenon, conflicting opinions have been reported in the literature on whether this behavioral pattern is to be considered a pathological addiction itself or, instead, an extreme of normal behavior that can take the form of problematic usage (Varona et al., 2022).

Intriguingly, while within the DSM-5 a diagnostic category for “internet gaming addiction” which focuses on the dysfunctional use of online gaming is available, no official diagnostic categories for internet addiction in general and social networking addiction are included, neither in the DSM-5 nor in the ICD-11. However, mounting evidence in the literature suggests that, as shown for other behavioral addictions such as gaming, excessive internet use in general and excessive use of social networks show numerous similarities with substance-based addictions. For example, typical psychological mechanisms associated with alcohol and drug addictions, such as withdrawal symptoms and tolerance, have been reported for internet and social media addiction as well (Bányai et al., 2017). Not only psychological but also cognitive features of substance use disorders have been reported in relation to internet and social media addiction. For example, alterations in executive functioning and inhibitory cognitive control have been reported in individuals suffering from social network addiction (Wegmann et al., 2020). Similarly, attention, as the most investigated cognitive domain subject to alterations in substance use disorders, has been shown to play a crucial role in internet and social media addiction as well, with clear evidence concerning the presence of attentional deficits and attentional bias (Jeromin et al., 2016; Wang et al., 2017; Nikolaidou et al., 2019).

Literature on this topic seems to suggest that being engaged in dysfunctional addictive behavior may serve as a coping strategy to manage emotional dysregulation because of stressful events that induce unpleasant emotions (Chou et al., 2015). While most of the available evidence focuses on contingent emotional states, less is known about the role of an individual tendency to be susceptible to certain emotions as a stable trait. This research line is mainly grounded in studies investigating the role of susceptibility to positive and negative affect in relation to mood and personality disorders (Larsen and Ketelaar, 1991) and studies investigating the relationship between general emotional susceptibility and interoceptive processes (Calì et al., 2015).

Furthermore, it should be noted that along with other negative consequences of the COVID-19 pandemic on personal general wellbeing (Cannito et al., 2020; O’Connor et al., 2021), relational phenomena (Cannito et al., 2022b), and economic and community organization (Cannito et al., 2021; Ceccato et al., 2021; Di Crosta et al., 2021), a consistent number of results suggests an increase in the prevalence of internet-based addictive behaviors (Masaeli and Farhadi, 2021) and smartphone misuse and separation anxiety (known as nomophobia) or dependency (Caponnetto et al., 2021). While this increase may be reasonably understood since technology use was the essential base of adaptability for smart working, schooling, and professional training, particularly during the strict lockdown phases, it remains unclear why some individuals continue to engage in these dysfunctional behaviors, even presenting the typical symptomatologic manifestations associated with addiction, including physiological and cognitive modifications (Konok et al., 2017). Intriguingly, since the beginning of the pandemic emergency, a consistent number of studies reported a significant increase in boredom experience among the population (Danckert, 2022) with fluctuations in levels of boredom associated with changes in the perceived passage of time during the lockdown phases (Wessels et al., 2022) and with boredom proneness predicting the violation of restrictive measures adopted by the governments (Boylan et al., 2021). While there is converging evidence suggesting a concomitant increase in internet addiction and the experience of boredom among the population, how emotional dysregulation associated with the experience of boredom promotes addictive behaviors remains an open question.

Following recent theorization suggesting the relevant role of attentional processes as core cognitive components of boredom (i.e., MAC Model; Westgate and Wilson, 2018), in the current study, we aimed to investigate the joint role of trait boredom (i.e., boredom susceptibility and the dispositional tendency to experience boredom) and altered attentive processing of relevant stimuli (i.e., Attentional Bias) in predicting internet addiction risk level.

### The role of boredom in addiction

Despite its theoretical significance as an indicator of psychological well-being and its prompting role in some human behavioral patterns, the emotion of boredom started to receive more structured attention from the psychology community only in recent years, probably due to the long-standing debate on boredom’s definition and nature (Fultz et al., 2022). According to the current literature, boredom can be defined as the subjective experience of being in a state perceived as undesirable and unpleasant (Eastwood et al., 2012), associated with a strong difficulty in maintaining attention and a tendency toward cognitive disengagement (Goetz and Hall, 2014; Elpidorou, 2018), as well as with a perceived slow passage of time (Witowska et al., 2020), which generally prompts people to take action to escape the present moment (Westgate and Wilson, 2018). Several models have been proposed to explain the emotion of boredom, most of which fall within three categories: attentional models, arousal/environmental models, and meaning/functional models of boredom. The first group (Eastwood et al., 2012) suggests that boredom results from a lack of engagement and attention to the task being performed. Therefore, when a task is perceived as uninteresting, it becomes difficult to sustain attention and focus, leading to boredom. For the arousal/environmental models (Cox, 1980; Chin et al., 2017), boredom is a result of low levels of physiological arousal and a lack of stimulation from the environment. Therefore, people who are bored are seeking new and exciting experiences to increase their level of arousal. According to meaning/functional models (van Tilburg and Igou, 2012), boredom is a result of a lack of meaning and purpose in an activity so when people feel that their actions are unimportant, they become bored and disengaged. Therefore, boredom’s function is to communicate the worthlessness of the current action in which the individual is involved (Westgate and Wilson, 2018). Among all of them, the model that has received the most support from experimental evidence is the MAC (Meaning and Attentional Components) model of boredom and cognitive engagement, according to which attention and meaning work as independent predictors of boredom and are both required to avoid the experience of boredom (Westgate and Wilson, 2018).

In addition to the literature investigating the nature of boredom from a theoretical point of view, in recent years, evidence has accumulated showing the possible positive and negative behavioral consequences induced by boredom. For example, it has been shown that creativity may serve as a cognitive coping strategy to reduce boredom that motivates an individual to pursue new goals, thus suggesting a positive contribution of boredom in promoting behaviors that improve an individual’s state (Elpidorou, 2018; Westgate, 2020). On the other side, boredom has also been shown to promote an individual’s involvement in undesirable behavior, such as an optimistic perception of risk and consequently increased risk-taking (Kılıç et al., 2020; Bench et al., 2021), or an increased risk of substance use disorders and addiction, particularly among the youngest (Biolcati et al., 2016; Yang et al., 2020; Donati et al., 2022).

While most of the available evidence on the causal impact of boredom on addiction pertains to state boredom as a negative transient emotion experienced in a specific situation, recent contributions suggest that trait boredom (also known as boredom susceptibility or boredom proneness in the literature) accounts for negative behavioral outcomes, particularly during the COVID-19 pandemic, independent of state boredom (Weiss et al., 2022).

Boredom as a trait refers to an individual’s stable tendency to easily experience boredom in several situations or activities. People who score high on measures of boredom proneness tend to find it difficult to be satisfied with their surroundings and may have a low tolerance for repetitive or unengaging experiences. It is important to note that trait boredom is a complex and multi-faceted trait that can be influenced by various individual, situational, and environmental factors. Tam et al. (2021) recently suggested that individual differences in trait boredom are reflected by differences in three macro-components: the frequency of getting bored, the intensity of boredom, and a holistic perception of life being boring, defined as perceived life boredom (Tam et al., 2021).

Following previous literature, it can be hypothesized that the level of trait boredom positively predicts levels of internet addiction.

### The role of attentional bias in addiction

The literature on cognitive correlates of addiction has long uncovered a very robust mechanism known as attentional bias (AB). AB manifests itself as a distortion of the normal processes that support selective attention, thus producing a strong tendency to direct attention toward the addictive stimuli (engagement phase) and/or difficulty in shifting focus away from such stimuli (disengagement phase). AB is commonly measured via a dot-probe task in which an addiction-related picture and a neutral picture are presented side by side (Lorenz et al., 2013). One of the two pictures is then replaced by a target (x) and participants are asked to indicate its position. In this case, people respond more quickly to the target if it appears in a most frequented spatial area surrounding one of the two pictures (Posner et al., 1980). As individuals suffering from an addiction respond more quickly to targets that replace addiction-related pictures, it has been suggested that they have heightened attention toward these stimuli (Field and Cox, 2008). Across addiction categories, this bias is considered to play an important role in the development and maintenance of dysfunctional addictive behavioral patterns. For internet- and social media-based addiction, it has been referred to as a tendency to pay more attention (both visive and auditive) to internet-related cues such as images of computer screens or notifications from social media, compared to neutral stimuli (Nikolaidou et al., 2019; Zhao et al., 2022). This bias in devoted selective attention is related to increased craving and internet use frequency and is also associated with differences in neural correlates. For example, an increased ERP-late positive potential to game-related stimuli in a sample of individuals with internet gaming disorder was reported (Kim et al., 2021). Additionally, studies have shown that attentional modification can be a pathway through which creating a psychological intervention for AB toward internet- and social media-related cues can be modified via attentional bias modification techniques, such as cognitive bias modification for addiction, which has shown promising results in reducing internet and social media craving and use (Xiaoxia et al., 2020; Camilla et al., 2022). Therefore, it was hypothesized that AB toward addiction-related stimuli may work as a positive predictor of internet addiction as measured in the current study.

## Materials and methods

### Participants

The sample included 70 (*N* = 13 men, mean age 19.42 ± 1.54 SD) Italian student participants. All the participants provided written informed consent in accordance with the ethical standards of the Declaration of Helsinki (1964). Participants were recruited through online public announcements and received no monetary or other compensation for their participation. To take part in the study, participants were required to be social network users and not to be diagnosed with any neurological or psychiatric condition. This information was self-reported by participants during the recruitment phase by answering two questions (1. Have you ever been diagnosed with a neurological or psychiatric condition? 2. Have you ever taken medication because of a neurological or psychiatric condition?). Exclusion from participation was determined by a positive response to either one or both questions. The whole experimental procedure was conducted in the laboratory and participants were instructed to perform the task and provide their answers to the questionnaires. For the visual dot-probe task, participants were asked to sit in front of a computer screen while maintaining a distance from the center of the screen of approximately 60 cm throughout the duration of the task.

### Measures

#### Internet addiction test

To measure participants’ risk level for internet addiction, we administered the Italian version of the internet addiction test, hereafter, IAT (Casale and Fioravanti, 2015; Servidio, 2017), adapted from the original scale (Young and Rogers, 1998). The scale includes 20 items on a 5-point Likert scale (from 1 = Never to 5 = Always) measuring the risk for internet addiction, with a possible score ranging from 0 to 100. Following Young’s original classification (Young and Rogers, 1998; Young and Case, 2004), a participant reporting a score above 30 should be considered at risk. The scale allows to individualize the risk level for addiction on four possible levels: severe risk (scores ranging from 80 to 100), moderate risk (scores ranging from 50 to 79), mild risk (scores ranging from 31 to 49), and no risk (normal usage, scores ranging from 0 to 30). Based on this classification and reported responses, our sample was distributed as follows: severe risk = 0%; moderate risk = 13.1%; mild risk = 69.5%; no risk = 17.4%. For our sample, Cronbach’s α for this scale was 0.86.

#### Trait boredom

To measure trait boredom, participants were administered the Italian version of the Brief sensation-seeking scale, hereafter, BSSS (Primi et al., 2011). The scale, developed as the shortest version of the Sensation-seeking scale (Zuckerman et al., 1978), allows four different factors to be measured, among which there is boredom susceptibility (BS-BSSS) consisting of an aversion to repetition and routine, and restlessness when things are not changing (Zuckerman, 1994). Participants are required to express their agreement with respect to eight items on a 5-point Likert scale (from 1 = Strongly Disagree to 5 = Strongly Agree). Based on our participants’ observed answers, the BSSS scale reports a Cronbach’s α = 0.89. Cronbach’s α for the boredom susceptibility subscale was 0.84.

#### Attentional bias toward social networks

##### Stimuli selection

A total of 80 pictures (20 social network logos, 20 brand logos, and 40 national flags), standardized for size and brightness, were selected from the web and administered to an independent sample (*N* = 35, mean age = 20.1 SD = 3.4 years old) in order to select 10 pictures highly associated with social networks (10 social network logos) and 30 pictures not associated with social networks (10 brand logos and 20 national flags). For this purpose, participants were asked to indicate how much, from 0 (not at all) to 100 (very much), the presented picture was associated with the idea of social network. The questionnaire was administered via Qualtrics software. Therefore, to construct the dot-probe task’s test trials, we selected the 10 social network logo pictures with the highest evaluation and the 10 brand logo pictures with the lowest evaluation. Similarly, to construct the filler trials, we selected the 20 national flag pictures with the lowest evaluation (for details see Supplementary Table 1).

##### Dot-probe task

**FIGURE 1:**
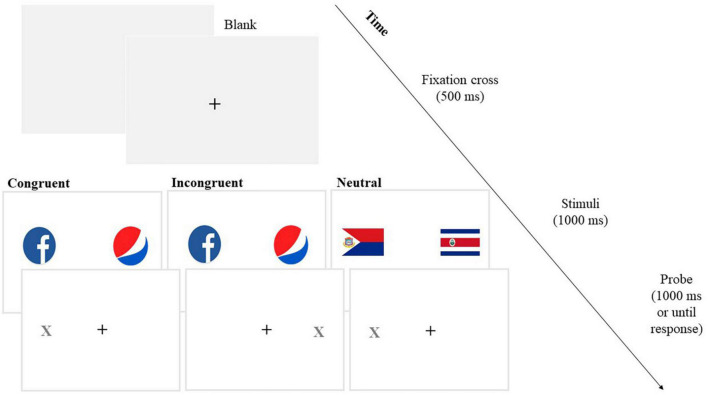
Dot-probe task.

To measure attentional bias toward social network stimuli, a modified version of the standard dot-probe task (Miller and Fillmore, 2010) was employed. The task involves the presentation of 10 pairs of social network/brand visual stimuli that were presented four times based on the four possible stimulus/probe combinations (the position of the stimulus on the left or the right and the position of the probe on the left or the right), thus obtaining 40 test trials. Also, there were 40 filler trials, which consisted of 10 pairs of neutral pictures (national flags) each presented four times. We included the filler trials in this task to reduce possible habituation to stimuli that might occur if all trials contained images related to the brands. The 40 filler trials were randomly intermixed among the 40 test trials, for a total of 80 trials. The task was divided into two blocks: the first block with 10 practice trials (for which geometric-shaped stimuli were employed to avoid possible familiarization effect with stimuli used for task trials) and the second block with 80 task trials (40 test trials and 40 filler trials), randomly sampled without replacement. After presenting the instructions, participants were presented with a fixation cross (+) at the center of the screen (500 ms), followed by the presentation of a couple of stimuli (social networks and brands pictures for test trials and both flag pictures stimuli for filler trials) showed for 1,000 ms. The position of the pictures was randomly chosen to be either on the left or on the right of the fixation cross. After that, the two stimuli disappeared, and a probe (X) appeared in the position of one of the two objects (the duration of the probe was 1,000 ms). Participants were asked to press one key (A) if the probe was on the left and another key (L) if the probe was on the right (see Figure 1). The task administration was conducted through a screen sized 15.6 inches and pictures were presented in a box of 6 × 7 cm (visual angle = 5.72° × 6.67°, calculated using a viewing distance of 60 cm) to the left and right sides of the centered fixation cross, with a distance of 10 cm between the two. Attentional bias is determined as a difference in the reaction times at congruent trials (trials at which the probe replaces the target stimulus, here the social network picture) and incongruent trials (the probe replaces the brand picture). For individuals whose attention is systematically drawn to the social network stimuli, reaction times are expected to be shorter (i.e., faster) for trials where the probe replaces the social network picture compared to trials where the probe replaces the brand picture.

## Results

Due to a technical error during task administration which prevented responses to the dot-probe task from being recorded, one participant was removed from the sample. The final sample included 69 participants (*N* = 13 men, mean age = 19.42 years ± 1.55 SD). A good accuracy percentage was found for all the types of trials: the trials’ accuracy when the probe replaced the target stimuli (i.e., social network logos) was 92.7%, and the trials’ accuracy when the probe replaced a neutral stimulus both from the test and filler trials (i.e., brand logo and national flags) was 91.1%. The overall accuracy was 92.9%. Before calculating attentional bias, some data filtering was performed. Trials with incorrect responses were not included in the dataset and reaction times shorter than 250 ms and longer than 1,000 ms were excluded. As a result, 89.3% of the original data were included in the following analyses.

Each participant’s mean reaction time per trial to probes was calculated for trial type (congruent versus incongruent). When considering test trials (no filler trials) in the whole sample, no significant RT difference was found between probes that replaced the target stimuli of social network logos (congruent trials, *M* = 360.94 ± 61.44 SD) and probes that replaced neutral images of brand logos (incongruent trials, *M* = 368.32 ± 68.32 SD), *t*(68) = −0.912, and *p* > 0.05. Pearson correlation coefficients were computed to assess the linear relationship between AB score, IAT score, and boredom susceptibility as obtained via BSSS. The results suggest significant positive correlations between all three variables (see Table 1 for details).

**TABLE 1:** Mean, standard deviations, and correlation coefficients between AB, IAT, and boredom susceptibility.

Variable	*n*	*M*	SD	1	2	3
1. AB	69	1.58	21.34	1	0.302*	0.308**
2. IAT	69	39.55	9.39		1	0.247*
3. Boredom susceptibility	69	6.43	1.74			1

**p < 0.01; *p < 0.05.

**FIGURE 2:**
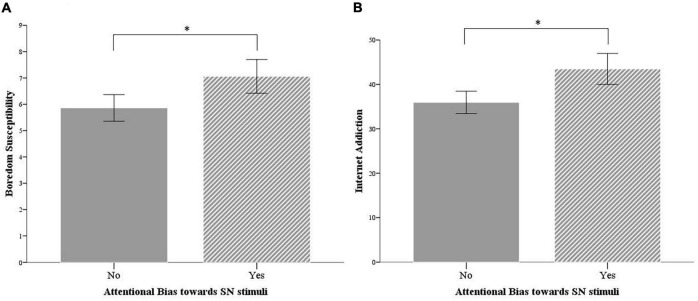
**(A)** Boredom susceptibility and **(B)** internet addiction for participants with and without attentional bias toward SN stimuli. Error bars, 95% CI. *p < 0.01.

Also, an independent sample *t*-test was performed to assess differences in IAT scores between participants that reported an AB (AB >0) and participants that did not report an AB toward social network stimuli (AB ≤0). The results indicated a significant difference in IAT scores, with significantly higher internet addiction levels for participants that showed AB (*N* = 33, *M* = 43.48, SD = 9.81) than for participants who did not (*N* = 36, *M* = 35.94, SD = 7.44), *t*(67) = 3.61, and *p* = 0.001, thus suggesting a significant contribution of an altered selective attentive process in internet addiction (see Figure 2A).

Similarly, participants who showed AB also presented a significantly higher level of boredom susceptibility (*N* = 33, *M* = 7.06, SD = 1.80), compared to participants who did not present AB (*N* = 36, *M* = 5.86, SD = 1.49), with *t*(67) = 3.018, and *p* = 0.004 (see Figure 2B).

To test the hypothesis that being more prone to boredom may increase the risk for internet addiction both directly and indirectly, through the intervention of an altered attentional engagement mechanism toward addiction-related stimuli, a mediation model was performed. As a first step, simple linear regression was used to test if boredom susceptibility significantly predicted the IAT score. The fitted regression model was: IAT = 30.98 + 1.33 (boredom susceptibility). The overall regression model was statistically significant, *R*^2^ = 0.061, *F*(1,67) = 4.36, and *p* = 0.04. Given the predictive role of boredom susceptibility on IAT score, a simple mediation analysis was conducted using the SPSS version (IBM SPSS, v. 22) of PROCESS macro and applying the Model 4, bootstrapping with 5,000 resamples to estimate indirect effects (Hayes and Preacher, 2013). This model is designed to test a situation in which the relationship between an outcome variable (IAT score) and a predictor variable (boredom susceptibility) can be explained by their relationship to a third variable (AB) named a mediator (Field, 2013).

**FIGURE 3:**
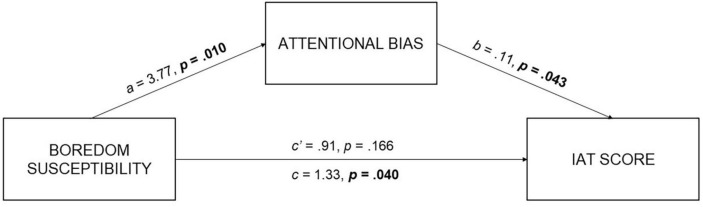
Mediation model. Significant *p*-values in bold.

Kappa-squared (κ^2^) value was calculated to measure the size of the indirect effect: a value around 0.25 indicates a large effect, a value around 0.09 indicates a medium effect, and a small effect value is expected to be around 0.01 (Field, 2013). For the proposed mediation model, a κ^2^ = 0.08 was computed (see Figure 3). As reported in Table 2, there was a full mediation of attentional bias toward social network stimuli on the relationship between trait boredom and internet addiction level.

**TABLE 2:** Mediation model predicting IAT score.

			95% CI	
**Effects**	**Path estimates**	**Coefficient (SE)**	**LL**	**UL**
The direct effect of BS-BSSS on AB	*a*	3.77 (1.42)**	**0.934**	**6.609**
The direct effect of AB on IAT	*b*	0.110 (0.053)*	**0.003**	**0.216**
The direct effect of BS-BSSS on IAT	*c*′	0.916 (0.654)	−0.390	2.223
The indirect effect of BS-BSSS on IAT	*ab*	0.414 (0.222)	0.092	1.013
The total effect of BS-BSSS on IAT (accounting for AB)	***c***	1.331 (0.637)*	**0.058**	**2.603**
	Total effect model: *F*(1,67) = 4.39 (*R*^2^ = 0.117*)			

BS-BSSS, boredom susceptibility; AB, attentional bias; IAT, internet addiction test; SE, standard errors; CI, bias-corrected and accelerated 95% confidence interval; LL, lower limit; UL, upper limit. A total of 5,000 bootstrap samples. Significant effects are in bold. Path coefficients are significant at **p < 0.01; *p < 0.05.

## Discussion

In the current study, attentional bias toward social networks has been identified as a mediator in the relation between trait boredom and internet addiction, suggesting that when someone chronically experiences boredom, their visual attention is more likely to be drawn to social media-related cues, increasing their risk of developing internet addiction. Indeed, mere exposure to addictive stimuli works as a factor that increases the risk of engaging in addictive behaviors. This is likely because social networks provide an easy form of entertainment and distraction from boredom, which can lead to a cycle of seeking out more and more online activities as a means of escape.

Our results are in line with a study by Al-Saggaf et al. (2019) which found that internet addiction, fear of missing out, and self-control were all related to trait boredom, finding that boredom proneness was a positive significant predictor of internet addiction. Our results are also in line with those obtained by Zhao et al. (2022) which suggest that problematic use of social media is associated with a higher attentional bias toward social media, and both are associated with a higher experience of negative emotions (anxiety, depression, social fear, and loneliness) even if the emotions examined were not limited to boredom (Zhao et al., 2022).

Similarly, evidence from another study indicated that boredom proneness in adolescents was linked to a wide range of risky behaviors, including internet addiction, binge drinking, problem gambling, and sexual activity during free time (Biolcati et al., 2018). The authors concluded that boredom proneness could be a significant risk factor for problem behaviors in adolescents and could be an important factor to consider when designing interventions to reduce risk by introducing new practices to manage free time, therefore working on the reduction of, at least, the frequency of getting bored between the three factors proposed as core components of trait boredom.

While our results work as corroboration of the existing literature as they support the idea that trait boredom may be a crucial element in defining a risk profile for internet addiction, they also add a new element to our understanding of the dynamic characteristics of this disorder. In particular, the evidence that the predictive role of trait boredom is fully mediated by the attentional bias toward disorder-relevant stimuli leads to at least two considerations. First, the idea that an individual stable trait’s influence on dysfunctional behaviors can be minimized by a more not stable and treatable cognitive characteristic is itself encouraging and promising concerning the investigation of intervention protocols for this disorder. Second, and more in need of further exploration, the idea that intervention protocols for reducing internet addiction should not focus exclusively on personality traits and affect modifications. Often, the structure of these interventions is strongly focused on the reduction of non-engagement and trait boredom through involvement in stimulation-type activities and particularly during free/leisure time (Waterschoot et al., 2021). However, our findings suggest that this may not be sufficiently effective if it is not accompanied by modification in cognitive alterations, such as those affecting the attentional system associated with the disorder itself. Taken together, and from a cognitive-behavioral perspective, most of the currently available interventions seem to focus on modulating behavioral aspects (e.g., associated with motor activation or avoidance reduction) while less attention has been paid to managing cognitive aspects. In this sense, an involuntary alteration of attentional focusing patterns on addictive stimuli may be interpreted as a dysfunctional coping strategy aimed at managing boredom when the perception of this emotion exceeds the threshold of tolerance. Therefore, it would be useful to further investigate this relationship and to test the modification of the coping strategy based on volunteer alteration of attentional focus as a possible therapeutic intervention.

The current study presents some limitations. First, since the instrument used in our study to measure the risk of internet addiction was developed several years ago, future studies should test the validity of this model using a more recent instrument for the assessment of internet addiction. Nevertheless, our results would be further strengthened by the presence of an objective measurement of internet addiction since our data on internet addiction, as self-reports, reflect the subjective perception of the participants. Future studies should consider testing the model using a different type of measurement, such as hours spent on the internet. Second, clinical interpretation of the current results should be done considering that no participant in the sample showed a severe risk for addiction to the internet (IAT >80) and a small portion (approximately 13%) presented a moderate risk of addiction (IAT = 50–79). Another limitation to be highlighted concerns the measurement of boredom susceptibility by means of two item-based factors that may not have captured all relevant aspects of trait boredom.

Moreover, future studies should amplify the investigation of the role of attentional bias as expressed through other sensorial channels (such as acoustic) and multisensorial attentive distortion, as possible different involvement of attentional distortion on different sensorial levels might vehiculate and help define subsequent intervention projection and testing. Nevertheless, the role of cognitive functioning and processing in domains other than the attentive one (such as memory, reasoning, or consciousness) should be taken into consideration when evaluating internet addiction. At last, it would be particularly interesting to explore if evidence obtained in the current study also applies to the older population (middle-aged and older adults) for which much less evidence is available in the literature on the prevalence and development of internet addiction.

Altogether, our results suggest that to reduce the risk of developing internet addiction, it is important to look for ways to cope with boredom other than social media, such as engaging in meaningful activities. Moreover, it is crucial to promote deeper integration of available knowledge on attentive processing of addiction-related information.

## Data availability statement

The raw data supporting the conclusions of this article will be made available by the authors, without undue reservation.

## Ethics statement

The studies involving human participants were reviewed and approved by the Local Ethics Committee Regione Abruzzo ASL 1 Protocol #0008934/20, 14/01/2020 int 271. The patients/participants provided their written informed consent to participate in this study.

## Author contributions

LC conceived the experiment and analyzed the data. EA, AB, and ED’I collected the data. LC and IC prepared the draft manuscript. All authors revised and approved the final manuscript.
