# Supplementary material for: Bored with boredom? Trait boredom predicts internet addiction through the mediating role of attentional bias toward social networks
Source: Front Hum Neurosci. 2023 Sep 1;17:1179142. doi: 10.3389/fnhum.2023.1179142 (PMC10513058; doi:10.3389/fnhum.2023.1179142)
Supplement: Supplementary file 1 [file Table_1.DOCX]

Supplementary Material

**B****ored with boredom? Trait boredom predicts internet addiction through the mediating role of attentional bias toward social networks.**

Loreta Cannito*, Irene Ceccato, Eugenia Annunzi, Alessandro Bortolotti, Eleonora D’Intino, Rocco Palumbo, Claudio D’Addario, Alberto Di Domenico, Riccardo Palumbo

*** Correspondence:**[loreta.cannito@unich.it](mailto:loreta.cannito@unich.it)

# Supplementary Table

**Table S1.** Mean score and standard deviation (SD) to the question: *“How much, from 0 (not at all) to 100 (very much), is this picture associated with the idea of social network?”*

| **Image Type** | **Trial** | **Mean score** | **SD** |
| --- | --- | --- | --- |
| Social Network Logo 1 | Test | 98.2 | 5.27 |
| Social Network Logo 2 | Test | 99.1 | 3.3 |
| Social Network Logo 3 | Test | 99.7 | 1.6 |
| Social Network Logo 4 | Test | 100 | 0 |
| Social Network Logo 5 | Test | 99.4 | 2.3 |
| Social Network Logo 6 | Test | 100 | 0 |
| Social Network Logo 7 | Test | 99.3 | 3 |
| Social Network Logo 8 | Test | 100 | 0 |
| Social Network Logo 9 | Test | 100 | 0 |
| Social Network Logo 10 | Test | 99.7 | 1.1 |
| Brand Logo 1 | Test | 0 | 0 |
| Brand Logo 2 | Test | 2.4 | 1.2 |
| Brand Logo 3 | Test | 0.9 | 2.3 |
| Brand Logo 4 | Test | 0 | 0 |
| Brand Logo 5 | Test | 0 | 0 |
| Brand Logo 6 | Test | 0 | 0 |
| Brand Logo 7 | Test | 6.5 | 5.8 |
| Brand Logo 8 | Test | 1.3 | 2.9 |
| Brand Logo 9 | Test | 3.2 | 4.2 |
| Brand Logo 10 | Test | 0 | 0 |
| Filler 1 | Filler | 0 | 0 |
| Filler 2 | Filler | 0 | 0 |
| Filler 3 | Filler | 0 | 0 |
| Filler 4 | Filler | 0 | 0 |
| Filler 5 | Filler | 3.0 | 4.5 |
| Filler 6 | Filler | 0 | 0 |
| Filler 7 | Filler | 0 | 0 |
| Filler 8 | Filler | 0 | 0 |
| Filler 9 | Filler | 0 | 0 |
| Filler 10 | Filler | 0 | 0 |
| Filler 11 | Filler | 0 | 0 |
| Filler 12 | Filler | 0 | 0 |
| Filler 13 | Filler | 0 | 0 |
| Filler 14 | Filler | 0 | 0 |
| Filler 15 | Filler | 0 | 0 |
| Filler 16 | Filler | 0 | 0 |
| Filler 17 | Filler | 0 | 0 |
| Filler 18 | Filler | 2.2 | 3.1 |
| Filler 19 | Filler | 0 | 0 |
| Filler 20 | Filler | 0 | 0 |
